# Aqueous Humor TGF-β2 and Its Association With Intraocular Pressure in a Naturally Occurring Large Animal Model of Glaucoma

**DOI:** 10.1167/iovs.64.10.18

**Published:** 2023-07-17

**Authors:** Kazuya Oikawa, Odalys Torne, David Sun, Alaina K. B. Moon, Julie A. Kiland, Ralph Møller Trane, Gillian J. McLellan

**Affiliations:** 1 Surgical Sciences, University of Wisconsin-Madison, Madison, Wisconsin, United States; 2 Ophthalmology and Visual Sciences, University of Wisconsin-Madison, Madison, Wisconsin, United States; 3 McPherson Eye Research Institute, Madison, Wisconsin, United States

**Keywords:** transforming growth factor beta (TGF-β), intraocular pressure (IOP), glaucoma, LTBP2, animal model

## Abstract

**Purpose:**

Transforming growth factor (TGF)-β2 has been widely implicated in human glaucoma pathology. The purpose of this study was to determine the source of TGF-β2 in aqueous humor (AH) and its relationship with intraocular pressure (IOP) in an inherited large animal model of glaucoma.

**Methods:**

Sixty-six glaucomatous cats homozygous for *LTBP2* mutation, and 42 normal cats were studied. IOP was measured weekly by rebound tonometry. AH was collected by anterior chamber paracentesis from each eye under general anesthesia, and serum samples collected from venous blood concurrently. Concentrations of total, active and latent TGF-β2 in AH and serum samples were measured by quantitative sandwich immunoassay. For comparisons between groups, unpaired *t*-test or Mann Whitney test were used, with *P* < 0.05 considered significant. The relationships between TGF-β2 concentrations and IOP values were examined by Pearson's correlation coefficient and generalized estimating equation.

**Results:**

IOP and AH TGF-β2 concentrations were significantly higher in glaucomatous than in normal cats. AH TGF-β2 showed a significant, robust positive correlation with IOP in glaucomatous cats (*r* = 0.83, R^2^ = 0.70, *P* < 0.0001). Serum TGF-β2 did not correlate with AH TGF-β2 and was not significantly different between groups. TGF-β2 mRNA and protein expression were significantly increased in local ocular tissues in glaucomatous cats.

**Conclusions:**

Enhanced, local ocular production of TGF-β2 with a robust positive association with IOP was identified in this spontaneous feline glaucoma model, providing a foundation for preclinical testing of novel therapeutics to limit disease-associated AH TGF-β2 elevation and signaling in glaucoma.

Glaucoma is a heterogeneous group of diseases characterized by a progressive, degenerative optic neuropathy leading to irreversible visual impairment, that is estimated to affect around 80 million people worldwide.^1^ Intraocular pressure (IOP) is the most consistent and major risk factor for development and progression of glaucoma.^2^^–^^4^ IOP is tightly regulated by the balance between aqueous humor (AH) production and drainage from the eye. AH is continuously produced at the ciliary epithelium and is drained from the anterior chamber of the eye to the systemic circulation, primarily through the conventional outflow pathway via the trabecular meshwork (TM).^5^^,^^6^ The unconventional outflow pathway via the ciliary muscle, supraciliary, and suprachoroidal spaces has been reported to contribute 4% to 55% of AH outflow in primates,^7^^–^^9^ but just 3% of AH outflow in cats.^10^^–^^12^ Disruption of normal structure and physiologic function of the AH outflow pathway results in damaging elevation of IOP in most forms of glaucoma.

We have previously established a viable colony of cats with a spontaneously occurring, recessively inherited feline congenital glaucoma (FCG),^13^^,^^14^ which is an ortholog of human primary congenital glaucoma (PCG) at the GLC3D locus (Online Mendelian Inheritance in Man #613086) due to a mutation in *LTBP2*, a gene encoding latent transforming growth factor-β binding protein 2 (LTBP2).^15^ Although *LTBP2* is known to be highly and preferentially expressed in anterior segment ocular tissues^16^^,^^17^ and known to play a role in extracellular matrix (ECM) assembly,^18^^,^^19^ the pathophysiology of glaucoma due to *LTBP2* mutations in humans remains unclear. Importantly, published studies have reported genetic variants or mutations in *LTBP2* associated with different forms of glaucoma, including PCG, juvenile open angle glaucoma (JOAG), and adult onset primary open-angle glaucoma (POAG),^20^^,^^21^ even within individual families. These studies suggest that LTBP2 could be involved in a wide range of glaucoma phenotypes. The feline model recapitulates many hallmarks of human glaucoma, including gradually progressive IOP elevation, following an initial onset early in life in this feline model,^22^ that is associated with progressive retinal ganglion cell (RGC) and optic nerve damage as well as functional deficits.^23^^,^^24^ Although the clinical phenotype of this feline glaucoma model has been rigorously characterized, the underlying aqueous outflow pathway pathophysiology has not yet been fully delineated in either this large animal model or in human glaucoma with *LTBP2* mutations.

Transforming growth factor-beta (TGF-β) is implicated in the pathogenesis of a wide range of ocular disorders, including glaucoma, playing pleotropic roles under normal and pathological conditions, particularly through its regulation of the amount and quality of ECM in ocular tissues.^25^^,^^26^ Among TGF-β isoforms (TGF-β1, -β2, and -β3), TGF-β2 is the predominant isoform in AH.^27^^,^^28^ There is ample evidence that dysregulated TGF-β signaling contributes to increased aqueous outflow resistance by altering ECM composition in the TM and leads to IOP elevation.^29^^–^^33^ A number of published independent studies and meta-analyses report higher TGF-β2 concentrations in AH samples from human patients with POAG than patients with cataract alone,^34^^–^^39^ further supporting the role of this cytokine in glaucoma pathogenesis. However, a direct association between AH TGF-β2 concentration and magnitude of IOP elevation was not definitively established in these human clinical studies. In addition, it has not been established whether systemic or local production (or both) of TGF-β2 contribute to the elevated levels of AH TGF-β2 observed in glaucoma.

To address these knowledge gaps, the current study was designed to test the hypotheses that AH TGF-β2 concentrations in glaucomatous cats would be higher than in normal cats, and that AH TGF-β2 would have a positive association with IOP in cats with glaucoma. As a secondary aim, we also determined whether serum TGF-β2 would be predictive of AH TGF-β2.

## Materials and Methods

### Animals

One hundred eight animals including 66 cats homozygous for *LTBP2* mutation causal for FCG (mean age ± SD = 1.5 ± 1.0 years; 32 male cats and 34 female cats) and 42 normal cats (1.9 ± 1.8 years, 22 male cats and 20 female cats) were studied. Animals were maintained under a consistent 12-hour light-dark cycle. All animal procedures were conducted in accordance with the Association of Research in Visual Science and Ophthalmology Statement on the Use of Animals in Ophthalmic and Vision Research, the National Institutes of Health (NIH) Guide for the Care and Use of Laboratory Animals, and in compliance with protocols approved by the Institutional Animal Care and Use Committee at the University of Wisconsin-Madison.

### Tonometry

Weekly IOP was measured by rebound tonometry (TonoVet; ICare Finland Oy, Vantaa, Finland) as previously validated, in awake, gently restrained cats.^40^ The animals were well acclimated to frequent tonometry since early in life. Three tonometer readings were acquired in accordance with manufacturer's instructions and averaged to provide a single value for each cat at each time point. All IOP values were obtained between 8 AM and 10 AM.^41^^,^^42^

### TGF-β2 Quantification

In total, 200 to 500 µL of AH were collected from each eye by anterior chamber paracentesis while the cat was under general anesthesia, and serum samples were collected from venous blood obtained concurrently, and both were stored at −80°C until subsequent analyses. Concentrations of TGF-β2 in AH and serum samples were determined by a quantitative sandwich immunoassay (Quantikine ELISA assay kit, catalog # DB250; R&D Systems, Minneapolis, MN, USA) following the manufacturer's protocol as validated for feline samples in our laboratory. As latent TGF-β2 is activated by freezing at −80°C,^43^ concentrations of TGF-β2 were measured prior to and following acid activation in a subset of 18 fresh, unfrozen AH samples, from 9 glaucomatous and 9 normal young adult cats (mean age of 2.54 ± 1.26 years, and 3.22 ± 1.27 years, respectively). Samples were stored on ice and assayed within 4 hours of sample acquisition. This enabled calculation of latent TGF-β2 from intrinsically activated and total (acid-activated) TGF-β2 concentrations. Optical density was determined by use of an automated microplate reader set at 450 nm (Multiskan EX Microplate Photometer; ThermoFisher Scientific; Waltham, MA, USA). Values were corrected for optical imperfections in the microplate by subtracting optical density values at 540 nm. Concentrations of TGF-β2 were determined from optical density values by comparison to a standard curve. All assays were performed in duplicate, and values were averaged for statistical analysis.

### Real-Time Quantitative Polymerase Chain Reaction

Total RNA was extracted from dissected, homogenized ocular tissues, as previously reported.^24^ Briefly, fresh lens, ciliary body, cornea, and sclera tissues from 3 normal young adult feline eyes were dissected under RNase-free conditions, and the dissected tissues were immersed in Allprotect Tissue Reagent (catalog # 76405; Qiagen, Hilden, Germany) and stored at -80°C, after overnight incubation at 4°C, until RNA extraction. Tissues were homogenized by TissueRuptor (catalog # 9002755; Qiagen) and total RNA was extracted using the RNeasy Fibrous Mini Kit (catalog # 74704; Qiagen) following the manufacturer's protocols. The RNAs were reverse-transcribed with GoScript Reverse Transcriptase and oligo-dT primers (catalog # A2791; Promega, Madison, WI, USA). TaqMan probes were designed and generated to target feline *TGFB2* (Forward: CAGAGTGCCCGAACAAAGGA, Reverse: GCTGGGTTGGAGACGTTAAGTC, Reporter probe: TCGAACTGTATCAGATTCTC) and *GAPDH* (Forward: GTGTCCGTCGTGGATCTGA, Reverse: GCTTCACCACCTTCTTGATGTCAT, Reporter probe: AAAGCTGCCAAATACG). Probe amplification efficacy was verified by serial dilution of feline cDNA. RT-qPCR was performed using TaqMan Fast Advanced Master Mix (catalog # 4444556; ThermoFisher Scientific, Waltham, MA, USA) and a QuantStudio 7 Flex Real-time PCR system (Applied Biosystems, Waltham, MA, USA) following the manufacturer's instructions. For relative quantification, the 2^−ΔΔCt^ method, implemented in Expression Suite software version 1.3 (ThermoFisher Scientific) was used. Results were presented as normalized to *GAPDH* expression.

### Immunofluorescence 

Globes from 5 glaucomatous and 5 normal age-matched, young adult cats (1-2 years old) were fixed in 4% paraformaldehyde overnight, and anterior segment tissues were dissected and processed for cryosections, as previously described.^44^ The tissue sections were immunofluorescence (IF) labeled following standard procedures. Briefly, the slides were blocked with 10% normal donkey serum in 0.01M PBS containing 0.2% Triton X-100 and incubated with anti-TGF-β2 antibodies (1:200, catalog # sc-90; Santa Cruz Biotechnology, Dallas, TX, USA) overnight at 4°C, followed by incubation with Alexa Fluor 568 conjugated donkey anti-rabbit secondary antibody (1:500, catalog # A10042; Life Technologies, Carlsbad, CA, USA) for 1 hour at room temperature, and nuclear counterstaining with DAPI (4′,6-diamidino-2-phenylindole). Each slide was washed then mounted with an anti-fade aqueous mounting medium (ProLong Gold; Life Technologies). For each batch of IF experiments, negative controls were included by substituting TGF-β2 primary antibody with rabbit IgG isotype controls (1:100, catalog # NBP2-36463; Novus Biologicals, Littleton, CO, USA). Ocular tissues known to express TGF-β2, such as the cornea, on each anterior segment tissue section served as positive controls. Mounted slides were examined and imaged using a Zeiss Axio Imager.Z2 microscope (Carl Zeiss AG, Oberkochen, Germany) with a 20× objective lens and identical image capture settings across slides and experimental batches. For IF signal quantification, background pixel intensity values were subtracted from IF signal pixel intensity values in a tissue region of interest. IF signal intensity values were averaged from three technical replicates per biological replicate and compared between groups.

### RNAscope In Situ Hybridization

In situ hybridization (ISH) was carried out using RNAscope Fluorescent Multiplex Kit V2 (catalog # 323100; Advanced Cell Diagnostics [ACD], Newark, CA, USA) according to the manufacturer's instructions with minor modifications. Briefly, 10 µm-thick tissue cryosections were air-dried for 30 minutes, washed in 0.01M PBS for 5 minutes, and treated with hydrogen peroxide for 10 minutes, all at room temperature, then incubated with protease IV for 30 minutes at 40°C using the HybEZ II Hybridization System (ACD). Tissue sections were incubated with feline specific probes targeting mRNA: *TGFB2* (catalog # 541521), *PPIB* (positive control, catalog # 455011), and *DapB* (negative control, catalog # 310043). Probes were fluorescently labeled with TSA plus Cy3 (Akoya Biosciences, 1:1500) and counterstained with DAPI. For ISH signal quantification, background and autofluorescence signals in images were removed using a blank image with Image J^45^ and RNAscope HiPlex Image Registration Software (ACD). ISH signal puncta were delineated and quantified using “subcellular spot detection” command on QuPath version 0.3.2.^46^

### Statistics

Statistical analyses were performed with Prism 8 (GraphPad, San Diego, CA, USA) unless otherwise stated. For the purposes of statistical analyses, mean IOP over 4 weeks prior to AH collection, and average of duplicate values of TGF-β2 were calculated. For comparisons between groups, unpaired *t*-test, Welch's *t*-test, or Mann-Whitney test were used as appropriate, with *P* < 0.05 considered significant. Normality was assessed by the Shapiro-Wilk test and Kolmogorov-Smirnov test. For linear regression analysis and Pearson's correlation coefficient, data from one eye per animal were used, due to high correlation between eyes (Pearson's correlation coefficient *r* = 0.95).^47^^–^^49^ For generalized estimating equation (GEE), IOP was modeled as a function of sex, genotype, age, and rescaled AH TGF-β2 concentrations, including an interaction effect between glaucoma genotype and AH TGF-β2 concentration. The model was fitted with animal identifier as variable, and an exchangeable correlation structure using the geepack package^50^ in R (version 4.2.0).

## Results

### Elevated IOP and AH TGF-β2 Concentrations in Cats With Glaucoma

**Figure 1.**
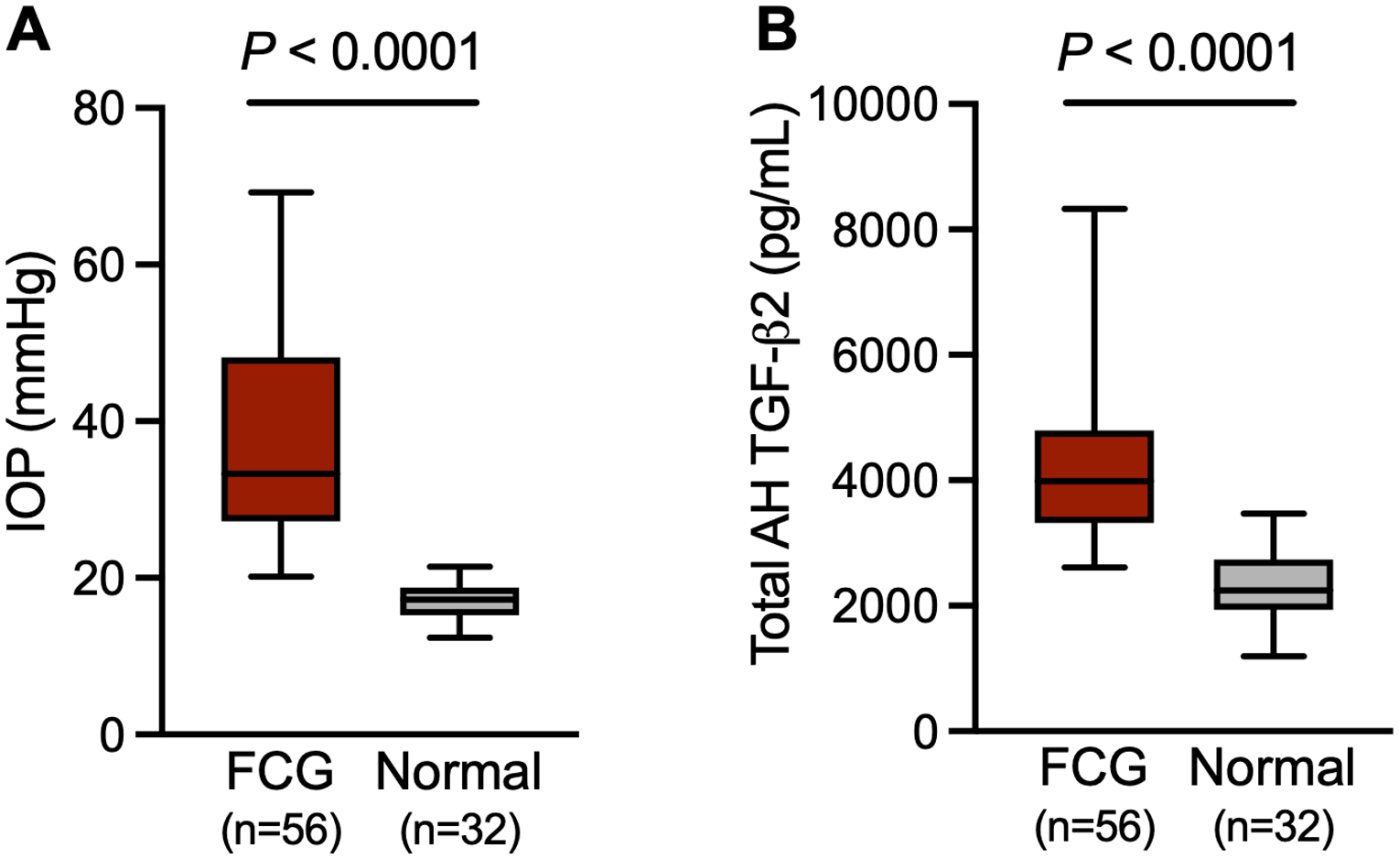
Intraocular pressure (IOP) and aqueous humor total concentrations of TGF-β2 were both elevated in cats with glaucoma relative to normal control cats. (**A**) IOP measured by rebound tonometry was significantly higher in young adult cats with feline congenital glaucoma (FCG; mean ± SD age = 1.1 ± 1.0 years old) than normal controls (mean ± SD age = 1.3 ± 1.6 years old; *P* < 0.0001, Mann-Whitney test). (**B**) Aqueous humor total TGF-β2 concentration in FCG was significantly higher than normal controls (*P* < 0.0001; Mann-Whitney test). (**A, B**) In each box and whisker plot, the median is shown as a horizontal line dividing the box which represents the interquartile range, and the whiskers show the 5th and 95th percentiles. Sample size for each group shown in the graphs.

Consistent with our previous study,^22^ IOP was significantly higher in FCG than normal controls (median values = 33.3 mm Hg and 17.3 mm Hg, respectively, *P* < 0.0001; Fig. 1A). Total AH TGF-β2 concentrations were significantly higher in FCG compared with normal control (median values = 3991 pg/mL and 2243 pg/mL, respectively, *P* < 0.0001; Fig. 1B).

### Elevated Active and Latent AH TGF-β2 Concentrations in Glaucomatous Cats

**Figure 2.**
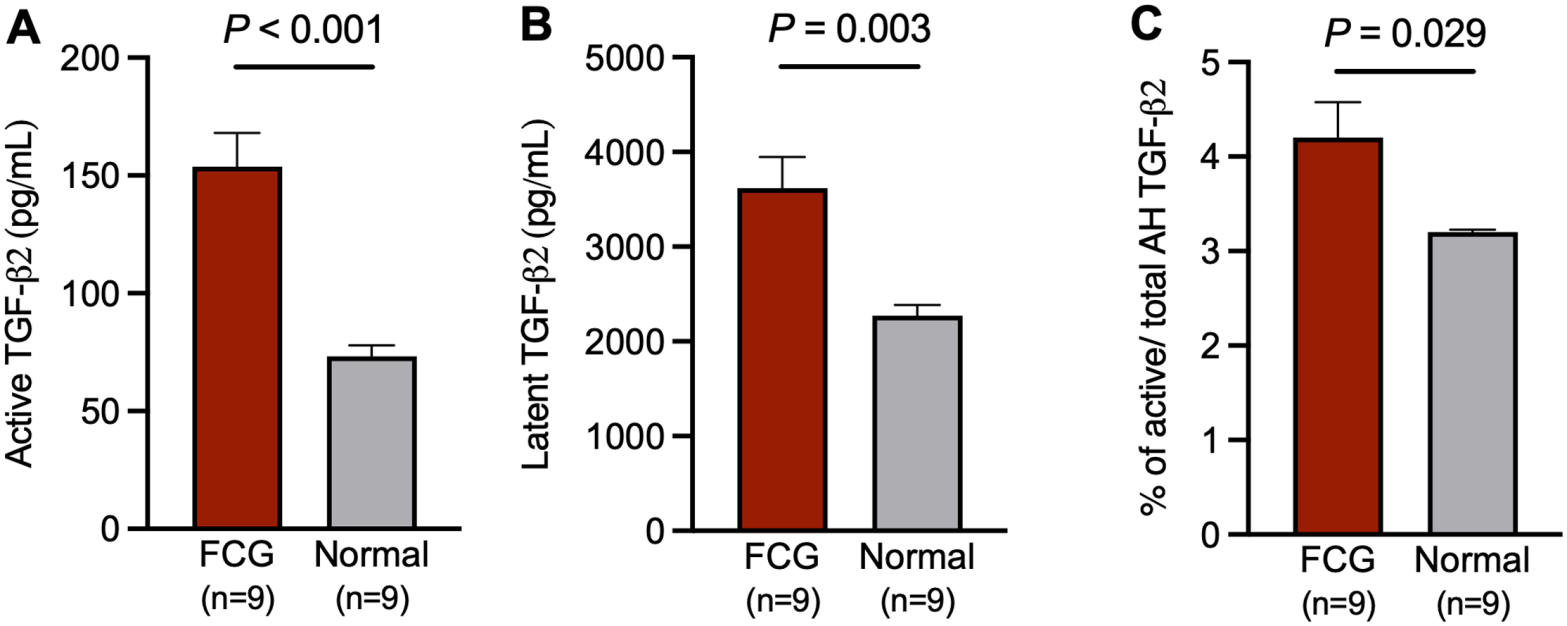
**Aqueous humor concentrations of both active and latent TGF-β2 are elevated in glaucomatous cats.** Active and latent TGF-β2 concentrations were measured in fresh AH samples from a cohort of FCG and normal young adult cats by ELISA. Active (**A**), latent (**B**), and the ratio of active to total AH TGF-β2 (**C**) were significantly higher in FCG compared to normal control (Welch's *t*-test). There were nine for each group and error bars represent SEM.

To determine if total, activated AH TGF-β2 concentrations identified in glaucomatous cats in the previous experiment represented increased TGF-β2 activation, we measured active and latent TGF-β2 in an additional cohort of 18 young adult animals. Mean (± SEM) concentration of intrinsically active TGF-β2 concentration in glaucomatous cats was 153.7 (± 14.3) pg/mL, compared with 73.28 (± 4.7) pg/mL in normal cats (unpaired *t*-test, *P* < 0.001; Fig. 2A). Latent TGF-β2 concentration was also significantly higher in glaucomatous cats (mean = 3620.0 ± 326.6 pg/mL) compared with normal cats (mean = 2274 ± 110.9 pg/mL; Welch's *t*-test, *P* = 0.003; Fig. 2B). Although the proportion of intrinsically active to total TGF-β2 in the AH of cats with glaucoma (mean = 4.2 ± 1.1%) was significantly higher than in normal control cats (mean = 3.2 ± 0.8%, Welch's *t*-test, *P* = 0.029), this difference was only modest, with the vast majority of AH TGF-β2 in both groups present in latent form (Fig. 2C). Thus, total, intrinsically active and latent TGF-β2 were all significantly increased in this feline glaucoma model relative to controls.

### AH TGF-β2 Concentration is Positively Correlated With IOP in Glaucomatous Cats

**Figure 3.**
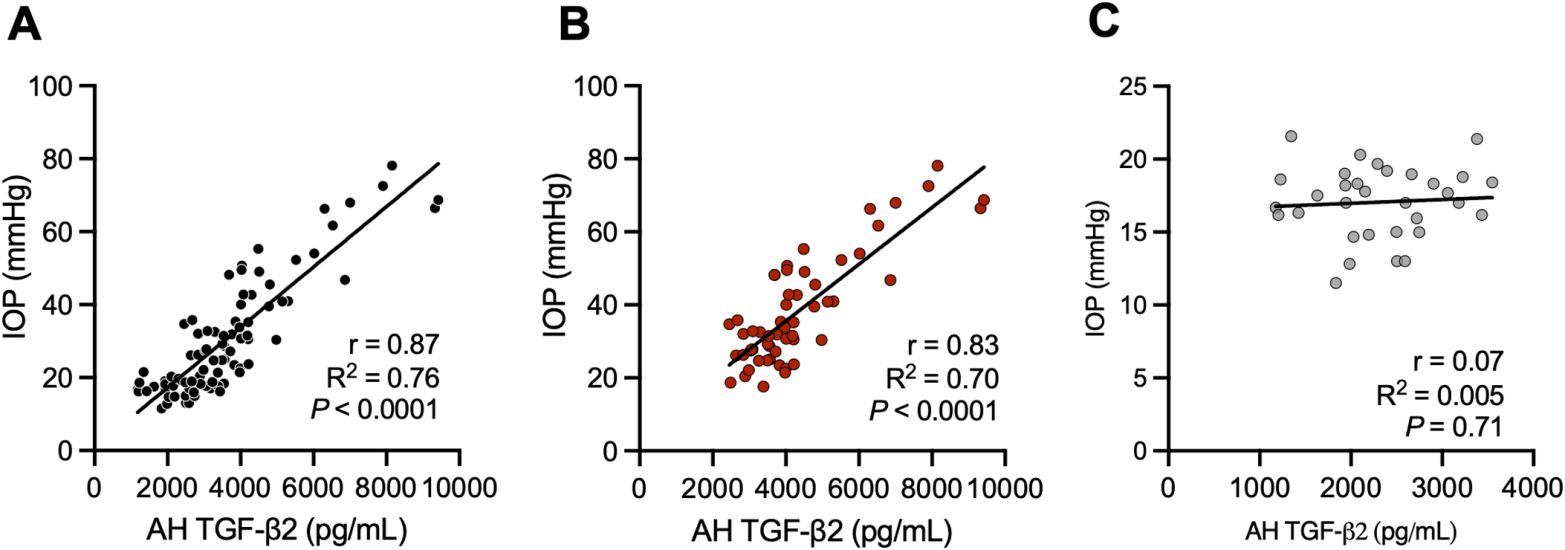
**TGF-β2 concentration in aqueous humor is strongly associated with IOP in glaucomatous cats.** Aqueous humor (AH) TGF-β2 concentration positively correlated with IOP for all animals analyzed collectively (**A**; *n* = 88). Whereas AH TGF-β2 demonstrated significant correlation with IOP in FCG (**B**; *n* = 56), no significant association was detected in normal cats (**C**; *n* = 32). A single measurement of AH TGF-β2 and mean IOP from each cat were included in these analyses.

We next sought to determine the association between IOP and AH TGF-β2 concentrations in glaucomatous cats. There was a significant positive correlation between AH TGF-β2 concentration and IOP for all cats analyzed (Pearson's *r* = 0.87, R^2^ = 0.76, *P* < 0.0001) and more specifically in cats with FCG (Pearson's *r* = 0.83, R^2^ = 0.70, *P* < 0.0001). No such correlation was identified for normal cats when analyzed separately (Pearson's *r* = 0.07, R^2^ = 0.005, *P* = 0.71; Figs. 3A–C). To further assess complex associations between IOP and AH TGF-β2, including factors of repeated measurements and high correlation between eyes from each cat, age and sex, we performed GEE analysis (see the Table). With a total of 229 AH samples included in the analysis, GEE identified that AH TGF-β2 concentration was significantly and positively correlated with IOP (estimate slope = 6.86, *P* < 0.0001) in cats with FCG, whereas no significant association between AH TGF-β2 and IOP was identified in normal cats (estimate slope = 0.072, *P* = 0.878), indicating that the effect of AH TGF-β2 on IOP is significantly different and greater in glaucomatous cats than in normal cats. Notably, there was no significant association between either sex or age and AH TGF-β2 concentrations in the cohort of young adult animals studied.

**Table.** Generalized Estimating Equation (GEE) to Assess The Association Between IOP and Aqueous Humor (AH) TGF-β2 For 229 Feline AH Samples

Variable	Estimate (95% CI)	*P* Value
(Intercept)	15.46 (12.08, 18.84)	<0.0001
Sex	1.76 (−1.19, 4.71)	0.241
Glaucoma	−5.53 (−8.91, 1.16)	0.236
AH TGF-β2 (ng/mL)	0.072 (−0.73, 0.88)	0.878
Age	0.019 (−0.034, 0.072)	0.48
**Glaucoma : AH TGF-β2 (ng/mL)**	**6.79 (4.52, 9.06)**	**<0.0001**

Analyses incorporated factors of repeated measurements and high correlation between eyes from each cat. GEE showed a highly significant positive correlation between AH TGF-β2 and IOP in cats with glaucoma (*P* < 0.0001). There was no significant interaction identified between either sex or age and AH TGF-β2.

CI, confidence interval.

### Serum TGF-β2 is Not Predictive of AH TGF-β2

**Figure 4.**
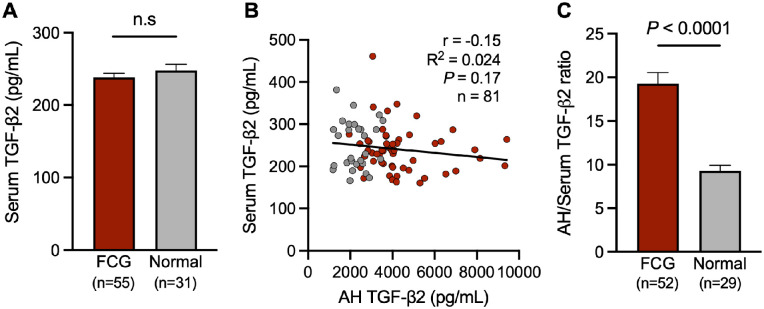
**Serum and aqueous humor TGF-β2 concentrations are not correlated in glaucoma.** Serum was collected concurrently at the time of aqueous humor (AH) sampling and total TGF-β2 in serum and AH quantified by ELISA. (**A**) No significant difference in serum TGF-β2 concentration was identified between FCG and normal cats (*P* = 0.29, unpaired *t*-test). (**B**) Serum TGF-β2 levels did not correlate with AH TGF-β2 levels in cats (*r* = −0.15, R^2^ = 0.024, *P* = 0.17, *n* = 82). (**C**) Ratio of AH to serum TGF-β2 concentration was significantly higher in FCG compared to normal controls (*P* < 0.0001, unpaired *t*-test). Each bar represents mean and error bars SEM. Sample sizes are shown in each graph.

To determine whether elevated TGF-β2 levels identified in AH reflect an underlying systemic dysregulation of TGF-β2 in glaucomatous cats with the *LTBP2* mutation, serum TGF-β2 concentrations were compared between glaucomatous and normal cats. Our results revealed no significant difference in serum TGF-β2 concentration between cats with glaucoma and age-matched controls (Fig. 4A). Further analyses revealed no correlation between serum TGF-β2 concentration and AH TGF-β2 concentration, either in all cats analyzed as a single group, or when analysis was confined to those cats with glaucoma (Fig. 4B). The ratio of AH to serum TGF-β2 concentration was significantly higher in FCG compared to normal cats (Fig. 4C). Together, these results demonstrate that serum TGF-β2 is not predictive of AH TGF-β2 and suggest local ocular production of TGF-β2.

### Enhanced Expression of TGF-β2 Transcript and Protein in Anterior Segment Tissues With Glaucoma

**Figure 5.**
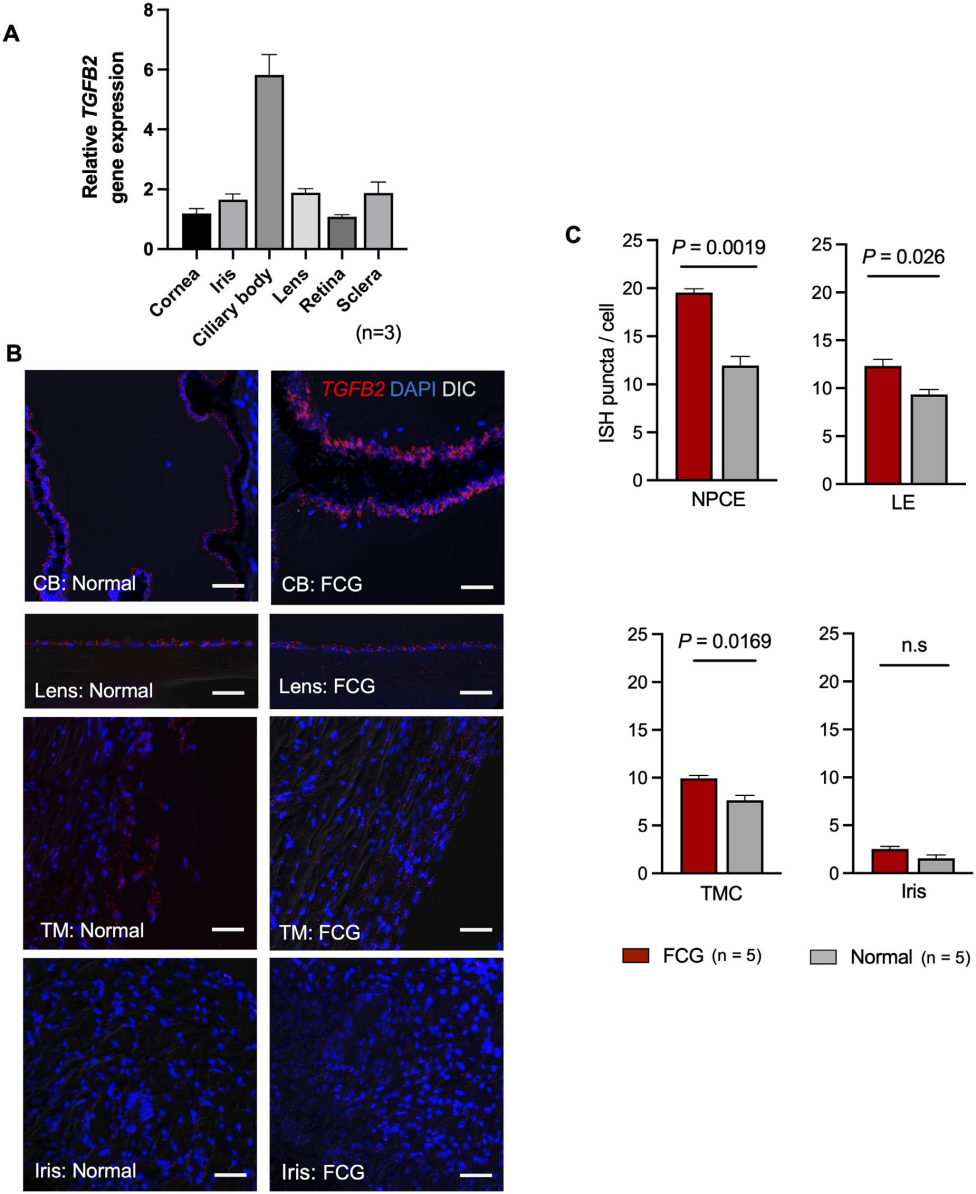
**Expression of TGF-β2 mRNA in feline ocular tissues.** (**A**) *TGFB2* transcript was highly expressed in the normal feline ciliary body. Transcript expression level by RT-qPCR in each ocular tissue is presented relative to the cornea. (**B**) Representative photomicrographs of the lens, ciliary body and iris in normal and FCG eyes with TGF-β2 transcripts visualized by RNAscope in situ hybridization (ISH) demonstrate enhanced expression of TGF-β2 mRNA in lens epithelial cells and non-pigmented ciliary epithelial cells in FCG*.* (**C**) TGF-β2 mRNA expression, assessed by quantifying ISH puncta per cell, was significantly greater in glaucomatous tissues and cells compared to age-matched normal controls (*n* = 5 per group). Each bar represents mean and error bars SEM. Scale bar = 50 µm. DIC, differential interference contrast; CB, ciliary body; NPCE, non-pigmented ciliary epithelium; LE, lens epithelium; TMC, trabecular meshwork cells.

As our data strongly supported local ocular TGF-β2 production, we next sought to identify major sources of TGF-β2 in ocular tissues. In normal adult feline ocular anterior segment tissues, the highest expression of *TGFB2* transcripts was identified by RT-qPCR in the ciliary body, followed by the sclera and lens (Fig. 5A). To further localize and quantify *TGFB2* transcript expression in situ, we performed RNAscope ISH which revealed enhanced *TGFB2* signals in lens epithelial cells, non-pigmented ciliary body epithelium, and TM cells of FCG cats relative to normal cats (Figs. 5B, 5C).

**Figure 6.**
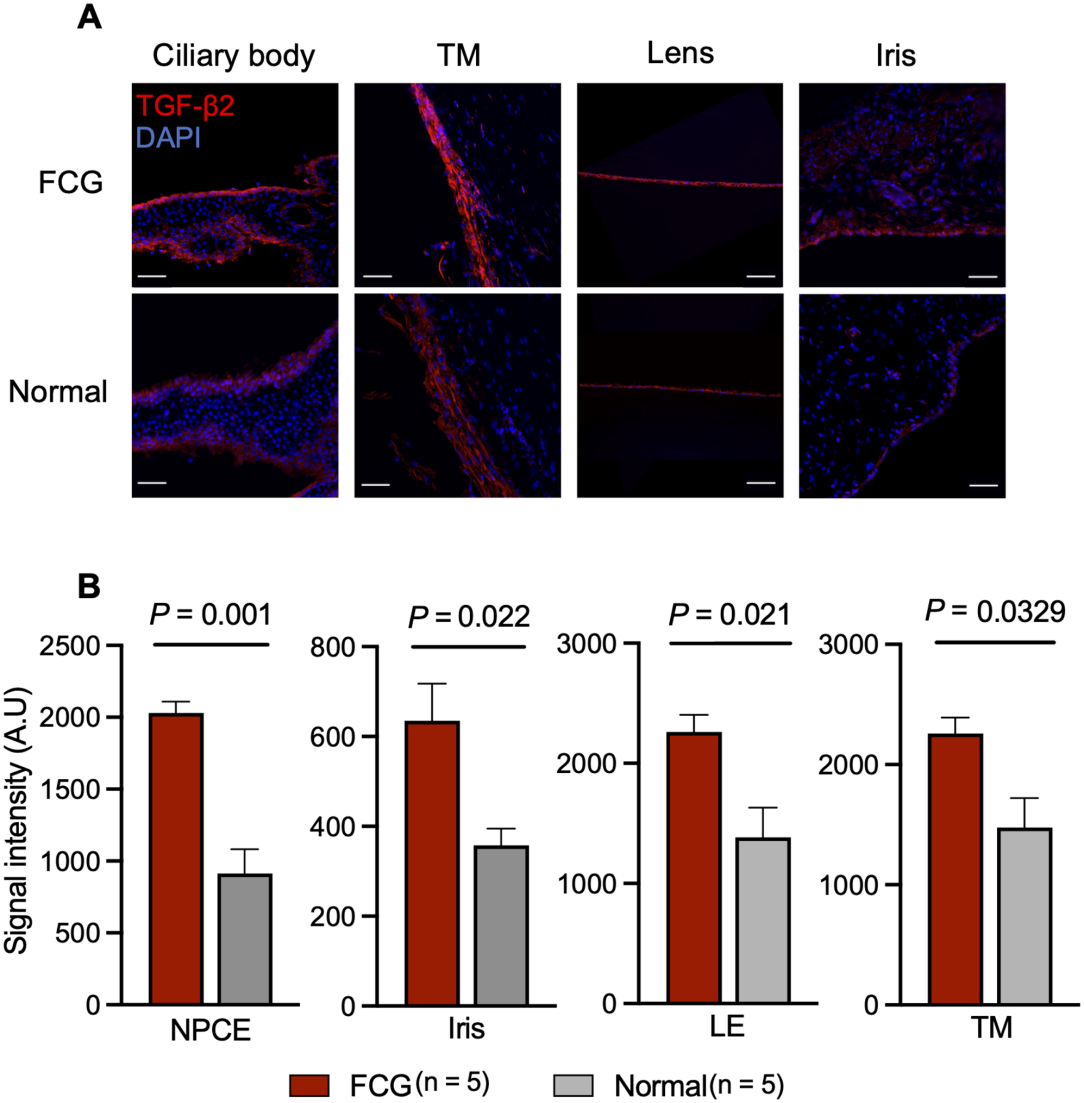
**Enhanced local ocular tissue TGF-β2 protein expression in glaucoma.** (**A**) Immunofluorescent (IF) labeling of TGF-β2 was performed on feline anterior segment cryosections. Representative photomicrographs of IF-labeled feline anterior segment sections demonstrate TGF-β2 immunoreactivity in the ciliary epithelium, trabecular meshwork (TM), lens epithelium, and iris in both normal and glaucomatous (feline congenital glaucoma [FCG]) eyes. (**B**) IF signal intensities of TGF-β2 in non-pigmented ciliary epithelium (NPCE), iris, lens epithelium (LE), and trabecular meshwork (TM) were significantly greater in FCG than in normal eyes. Scale bar = 50 µm. Each bar represents mean and error bars SEM. There were five biological replicates per group.

Next, we examined TGF-β2 protein expression in feline ocular tissues by immunofluorescence labeling. TGF-β2 was highly expressed in the trabecular meshwork, lens epithelium, and ciliary body epithelium in cats (Fig. 6A). Quantitative assessment of TGF-β2 expression by fluorescence intensity supported higher TGF-β2 expression in glaucomatous tissues (TM, iris, ciliary body epithelium, and lens epithelium) compared to normal controls (Fig. 6B). Collectively, these results suggest that lens epithelial cells and ciliary body epithelial cells are major local ocular sources of the increased TGF-β2 concentrations identified in AH of glaucomatous cats.

## Discussion

In the current study, we identified elevated TGF-β2 concentrations in AH in a naturally occurring feline glaucoma model. Notably, we directly demonstrate a robust, positive association between AH TGF-β2 and IOP in vivo in spontaneous glaucoma, in the absence of potentially confounding ocular medical or surgical treatments and/or other ocular diseases. Additionally, this study confirms that elevated AH TGF-β2 concentrations are attributable to enhanced local ocular TGF-β2 expression and have no association with circulating TGF-β2 concentrations in serum. The ciliary body epithelium, lens epithelium, and TM cells are major sources of AH TGF-β2. These findings strongly support a role for AH TGF-β2 in sustained IOP elevation in this translationally relevant feline model, as suggested in human glaucoma and experimental models.

A substantial body of literature has consistently shown higher TGF-β2 concentrations in the AH of human patients with POAG.^34^^–^^39^^,^^51^ However, human patients with glaucoma in these studies had potential confounders, including varying glaucoma medications and cataract. A direct statistical association between AH TGF-β2 and IOP has not been definitively established previously in either human patients with glaucoma or animal models, whereas weak to moderate associations between the other TGF-β isoforms (TGF-β1 and -β3) in AH and IOP have been reported in human patients with POAG.^36^ Besides POAG eyes, elevated total AH TGF-β2 has been reported in other forms of glaucoma, including primary angle closure glaucoma^35^^,^^52^ and neovascular glaucoma,^53^ although the underlying pathophysiology is likely different between these various forms of glaucoma in humans. Further, elevated AH TGF-β2 level has also been reported in a glucocorticoid-induced ocular hypertension model in rodents^54^ and microfibril-deficient tight skin (Tsk) mice with glaucoma-like phenotype.^55^ These studies support a role for TGF-β2 in the pathology of various types of glaucoma.

The finding of elevated AH TGF-β2 concentrations in this feline model, although generalizable to other forms of glaucoma, may be most directly relevant to human glaucoma due to *LTBP2* mutations, contributing insight into the pathology of this complex, poorly understood genetic disease. Our results demonstrated that the *LTBP2* mutation in glaucomatous cats has minimal effects on systemic TGF-β2 concentrations as serum TGF-β2 concentrations in *LTBP2* mutant cats were not significantly different than in normal control cats. It has been shown that LTBP2 does not covalently bind to TGF-β like the other LTBPs,^56^ suggesting little *direct* effect of *LTBP2* null mutations on TGF-β2 concentrations or activation status. Importantly, the proportion of TGF-β2 that was intrinsically activated relative to total TGF-β2 was not substantially altered in the AH of *LTBP2* mutant cats. Published single-cell studies showed that *LTBP2* is highly expressed in trabecular meshwork cells and non-pigmented ciliary epithelium in humans,^16^^,^^17^ with minimal transcript expression of *LTBP4* which compensates for loss of *LTBP2* in microfibril formation in non-ocular tissues, such as the lungs and aorta.^57^ Thus, it is conceivable that microfibril abnormalities caused by the loss of function mutation in *LTBP2* lead to increased ocular TGF-β2 expression in this feline model.

Specific mechanisms by which elevated AH TGF-β2 modulates IOP in vivo in spontaneous glaucoma remain elusive and are likely complex. To delineate mechanisms of IOP elevation in this feline model, more detailed morphological, ultrastructural, and molecular analyses addressing the effects of TGF-β2 on aqueous outflow pathways in normal and glaucomatous cats, including studies in a younger cohort of cats, and in TM cells in vitro, are currently underway in our laboratory.

The mechanistic roles of TGF-β2 in ocular hypertension and glaucoma have been extensively studied in vitro, in vivo, and ex vivo across species. A body of evidence from studies on cultured human TM cells in vitro indicate that TM cells treated with TGF-β2 exhibit altered expression of ECM proteins, and cross-linked actin network formation.^31^^,^^32^^,^^58^^–^^60^ TGF-β2 regulates the expression of ECM proteins in TM cells through the canonical SMAD and noncanonical signaling pathways.^61^^–^^64^ In ex vivo human and porcine anterior segment perfusion culture models, TGF-β2 is shown to reduce outflow facility and increase IOP, corresponding to accumulation of ECMs in the TM.^29^^,^^30^^,^^65^ In addition, overexpression of biologically active human TGF-β2 by viral vector gene transfer in rodent eyes induce ocular hypertension,^66^^–^^69^ via TGF-β2 signaling through the canonical SMAD pathway.^64^ Collectively, these studies provide compelling evidence that overactivation of TGF-β signaling modulates IOP by altering the quantity and quality of ECMs in aqueous outflow pathways, including the TM, increasing outflow resistance, which ultimately leads to IOP elevation. Thus, even secondary increases in TGF-β2 concentrations can modulate ECM composition, AH outflow, and IOP, most consistently reported in POAG and can also negatively impact outcomes of glaucoma surgery, such as trabeculectomy.^38^^,^^70^^,^^71^ Although a robust, positive association between AH TGF-β2 levels and IOP was identified in cats with glaucoma, the present study was not designed to establish the cause-and-effect relationship between these factors. Accumulating evidence from in vivo and ex vivo studies support that TGF-β2 contributes to IOP regulation and dysregulation.^29^^,^^30^^,^^68^^,^^69^^,^^72^ It is also conceivable that IOP-mediated signaling in ocular tissues could result in increased AH TGF-β2 levels in glaucoma.

AH concentrations of TGF-β2 far exceeded serum concentrations, supporting local ocular production versus an effect of blood aqueous barrier breakdown, and intraocular tissues that have contact with AH clearly are major contributors to AH TGF-β2 levels. These include TM cells and, to an even greater extent, ciliary body and lens epithelia, which express TGF-β2 transcripts and secrete TGF-β2 proteins.^73^^,^^74^ Although increased AH concentrations of secreted proteins in glaucoma could conceivably reflect reduced egress from the eye via the compromised aqueous outflow pathways, we observed increased intensity of mRNA expression and TGF-β2 immunolabeling of non-pigmented ciliary and lens epithelia consistent with enhanced local production of the cytokine, rather than simply reduced outflow. Importantly, we identified the tissues responsible for its local production, to inform future targeted therapeutic interventions in our translationally relevant large animal model.

The higher expression of TGF-β2 mRNA in situ in the lens epithelium and non-pigmented epithelium of the ciliary body in glaucomatous cats, further supports a role for these tissues as a major source of increased AH TGF-β2 levels in glaucoma. Thus, these ocular tissues represent important target sites for therapeutic intervention to limit elevation of AH TGF-β2 in glaucoma. Subtle increase in TGF-β2 protein expression noted in the iris in the absence of significant increase in TGF-β2 mRNA. However, TGF-β2 mRNA and protein expression in the iris was lower relative to the other ocular tissues examined, thus the iris likely does not represent a major source of the cytokine.

Important ethical considerations in the current study, common to all studies involving “large” animal models, limited our investigations to minimally invasive procedures for most of the animals involved, with only a small number of eyes used for postmortem experiments. However, the small sample size provides initial proof-of-concept evidence for the source of AH TGF-β2 in eyes with glaucoma. The cause-and-effect relationship between AH TGF-β2 and IOP in this feline model will need to be determined in future studies involving a younger animal cohort prior to detectable and significant IOP elevation. Additionally, the effect of *LTBP2* mutation on the expression of various ECM proteins in the AH outflow pathways will be explored in ongoing in vivo, in situ, and in vitro experiments.

## Conclusion

In conclusion, this study demonstrates elevated AH TGF-β2 concentrations in a spontaneous, translationally relevant large animal model of glaucoma consistent with earlier reports in AH samples from human patients with POAG. Robust association between AH TGF-β2 and IOP support a key role played by TGF-β2 in progressive IOP elevation identified in our model. Our results indicate that serum TGF-β2 is not a valuable biomarker for glaucoma screening and confirm local ocular production of TGF-β2, with the ciliary and lens epithelia as major sources of TGF-β2 in the AH and anterior segment tissues. Our findings provide a foundation for development and preclinical testing of novel therapeutic strategies to limit pathological AH TGF-β2 elevation and signaling in glaucoma.
